# Sociodemographic and Marital Differences in Spousal Involvement in a Partner’s Diabetes Diet

**DOI:** 10.1093/geroni/igaa057.1467

**Published:** 2020-12-16

**Authors:** Paulene Castro, Kristin August, Dara Sorkin

**Affiliations:** 1 Rutgers University-Camden, Oaklyn, New Jersey, United States; 2 Rutgers University, Camden, New Jersey, United States; 3 University of California, Irvine, Irvine, United States

## Abstract

Spouses are commonly involved in their partners’ diabetes management by supporting and regulating (i.e., controlling) their diet. Little is known, however, about what characteristics are associated with how and how often spouses are involved in this context. This study examined whether sociodemographic and marital characteristics helped explain some of the variability in diet-related spousal involvement in promoting a partner’s adherence to a diabetes diet; specifically, whether gender, race/ethnicity, marital quality, and marital length were related to the frequency of spousal engagement in health-related social support and two types of health-related social control. Gender and race/ethnicity were examined as exploratory moderators of the associations between marital characteristics and spousal involvement. Data from two different data sets of older adults (55+ years) whose partners had type 2 diabetes were examined among four racial/ethnic groups (study 1 n = 205; study 2 n = 155). Regression analyses that controlled for patients’ co-morbid health conditions revealed gender and racial/ethnic differences in the frequency of spousal involvement. In addition, marital quality was related to the frequency of support and positive forms of social control among most participants, particularly African American spouses. No associations between marital length and any type of spousal involvement were found, nor were there any gender differences in any of these associations. These findings provide insight into the importance of sociodemographic characteristics and marital quality in understanding spousal involvement in a partner’s diabetes management.

